# Novel Sodium Hypochlorite Cleanser Shows Clinical Response and Excellent Acceptability in the Treatment of Atopic Dermatitis

**DOI:** 10.1111/pde.12150

**Published:** 2013-04-26

**Authors:** Caitriona Ryan, Richard E Shaw, Clay J Cockerell, Shari Hand, Fred E Ghali

**Affiliations:** * Department of Dermatology, Baylor University Medical CenterDallas, Texas; † Clinical Informatics and Reporting Quality Department, Division of Cardiology, California Pacific Medical CenterSan Francisco, California; ‡ Department of Dermatology, University of Texas SouthwesternDallas, Texas; ¶ Top MD Skin Care IncDallas, Texas

## Abstract

The intermittent use of dilute sodium hypochlorite “bleach baths” has shown efficacy as adjunctive therapy for atopic dermatitis (AD). This feasibility study evaluated the clinical response and patient acceptability of treatment with a cleansing body wash containing sodium hypochlorite in children with AD. This was a 12-week open-label feasibility study of 18 children with AD conducted in a pediatric dermatology outpatient clinic between May 2011 and July 2012. Children with moderate to severe AD, defined as an Investigator Global Assessment (IGA) score of at least 3 on a 5-point scale, who were age 6 months and older and had lesional cultures positive for *Staphylococcus aureus* at baseline were included. Patients were instructed to wash 3 days/week for 12 weeks with the sodium hypochlorite–containing cleansing body wash. During the study period, patient's individualized topical and systemic treatment regimens were continued. Clinical response to treatment was measured using an IGA score and the percentage of body surface area (BSA) affected. Parents were also administered a retrospective questionnaire evaluating acceptability of the product. There was a statistically significant reduction in IGA score at all time points, with an overall mean reduction from baseline to final measurement using the last observation carried forward in all patients of 1.0 (p = 0.001, *n* = 18). Similarly the mean reduction of BSA affected was 14.8% (p = 0.005, *n* = 18). Parents reported that the body wash was significantly easier to use than traditional bleach baths (p < 0.001). The significant reductions in clinical disease severity scores with use of this formulation are encouraging.

Atopic dermatitis (AD) is a chronic immune-mediated disease of cutaneous inflammation and barrier dysfunction that affects approximately 10% to 20% of children worldwide 1,2. The effect of AD on a child's everyday psychological, emotional, and social functioning is considerable and, alongside the cost of long-term treatment, creates a significant social and economic burden 3. The marked susceptibility of individuals with AD to colonization and infection of the skin with *Staphylococcus aureus* is thought to play a significant role in exacerbation and maintenance of the disease 2,4–6. *S. aureus* is isolated from lesional skin in up to 90% of patients, and a correlation exists between disease severity and bacterial load 7–10. Superantigens that strains of *S. aureus* colonizing AD skin produce inhibit the activity of T regulatory cells that normally suppress inflammation, generating a state of resistance to topical corticosteroid therapy and triggering disease exacerbations 11. Moreover, with the alarming increase in the prevalence of multidrug-resistant *S. aureus,* particularly methicillin-resistant *S. aureus* (MRSA) 12, alternative methods are being sought to reduce the frequency of oral antibiotic therapy to counteract the growing epidemic of antibiotic resistance in the general community.

Several other antiseptic formulations have been investigated for use in AD. Resistance rates of MRSA to topical antibiotic agents such as mupirocin and sodium fusidate are increasing rapidly 13,14. Triclosan is a chlorinated aromatic compound with antibacterial and antifungal properties, but reports of triclosan resistance and the possibility that this may contribute to antimicrobial resistance through cross-resistance or coresistance mechanisms is of concern 15. Chlorhexidine is an antiseptic that is bactericidal and bacteriostatic against Gram-positive and Gram-negative bacteria, with activity against fungi and enveloped viruses. It has the significant disadvantage of being contraindicated for use near the eyes and ears because it has toxic effects on the cornea and can cause sensorineural deafness after direct instillation into the middle ear. It may also cause significant skin irritation.

Sodium hypochlorite has been used in clinical practice for more than 70 years for a multitude of infectious indications because of its bactericidal, antiviral, and sporicidal properties. It has been widely accepted that the use of sodium hypochlorite does not lead to the development of antimicrobial resistance. Unlike other antiseptic preparations, dilute sodium hypochlorite has been found to be nontoxic to tissues and mucosal surfaces in human and animal studies 16,17. Many dermatologists now incorporate intermittent use of dilute sodium hypochlorite “bleach baths” as an adjunctive therapy for individuals with AD. Although one study showed a significant reduction in AD disease severity with this regime 18, controversy exists as to the interpretation of these results 18,19.

The primary objective of this pilot study was to evaluate the clinical response to treatment with a cleansing body wash containing sodium hypochlorite (CLn BodyWash; Top MD Skin Care Inc., Dallas, TX) in patients with moderate to severe AD. Change in quantitative bacterial load from baseline, tolerability of the product, and patient adherence and satisfaction were examined as secondary endpoints.

## Materials and Methods

This was a 12-week, single-center, open-label, partially retrospective, partially prospective, nonrandomized feasibility study of 18 children with AD conducted between May 2011 and July 2012 in Dallas, TX. Eleven of 18 patients had data collected retrospectively, and the remaining 7 had data collected prospectively in a standardized manner (at baseline and weeks 4, 8, and 12), once the beneficial effects of this new topical regime became apparent. The Schulman Associates Institutional Review Board, Cincinnati, Ohio, granted ethical approval (IRB number 201104754/201104755). A parent or guardian of each child gave informed written consent. Patients with moderate to severe AD, defined as an Investigator Global Assessment (IGA) score of at least 3 on a 5-point scale, who were age 6 months or older and had positive *S. aureus* lesional cultures at baseline were included in the study. Exclusion criteria included clinically active infections (abscesses, boils, or purulent discharge) and topical or systemic antibiotic use in the previous 2 weeks. Information on patient demographic characteristics and clinical disease severity, including baseline IGA score and percentage body surface area (BSA) affected, were recorded. One or two bacterial cultures were taken at baseline from the worst affected lesional site(s) of AD. (A positive *S. aureus* culture was a prerequisite for entry to the study.)

Individuals who met the aforementioned criteria were instructed to wash 3 days/week for 12 weeks using the sodium hypochlorite–containing cleansing body wash. Patients lathered with the body wash from the neck down and then rinsed it off after 1 to 2 minutes. During the study period, patient's individualized topical and systemic treatment regimens were continued, and any treatment modifications were recorded. After the initial follow-up visit, patients were given the option to advance to daily usage with the body wash if desired. The same clinician recorded the IGA score, BSA affected, and adverse events for each patient at all visits. Because the follow-up time points were not standardized in this exploratory study, follow-up intervals were used to group data at weeks 2 to 4, 4 to 6, 6 to 8, and 10 to 12. Repeat bacterial cultures were obtained from the same baseline culture site at each visit. Cultures were quantified at Quest Diagnostics and Laboratory Corporation of America (Dallas, TX), with bacterial load measured crudely (0 = no growth, 1 = scant growth, 2 = mild growth, 3 = moderate growth, 4 = heavy growth). When two cultures were taken, the mean value was used for each patient. After termination of the study, parents were administered a retrospective questionnaire evaluating the acceptability and tolerability of the product. The questionnaire addressed adverse effects such as skin irritation or burning, ease of administration, and overall satisfaction with the product. For the 13 patients who had previous experience with traditional dilute sodium hypochlorite “bleach baths,” similar questions, including their preference of regimen, were ascertained.

## Results

Of the 21 patients screened to participate, 18 were recruited to the study. One of the screened patients showed no growth on initial culture, and the other two required oral antibiotic therapy on the day of screening. Baseline characteristics are summarized in Table 1. There were 7 boys (39%) and 11 girls, with a mean age of 9 years (range 2–11). Mean baseline IGA was 3.5 (range 3–5), and mean baseline BSA affected was 49.5% (range 15%–93%). Mean *S. aureus* quantitative bacterial culture count was 3.11 (range 1–4). Eight of the 18 enrolled patients (44%) had positive skin cultures for MRSA during the study period.

**TABLE 1:** Demographic Data at the Baseline Clinical Assessment

Characteristic	Value
Male, *n* (%)	7 (39)
Age, years, mean (range)	9 (2–11)
Baseline IGA, mean (range)	3.5 (3–5)
Baseline body surface area, %, mean (range)	49.5 (15–93)
*S. aureus* bacterial culture quantitative load	3.1 (1–4)

**Figure 1:**
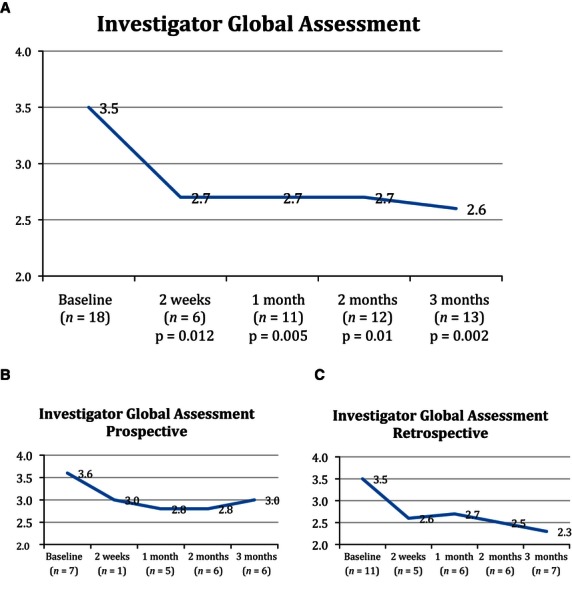
Mean change in IGA score from baseline visit to final visit in the (**A**) overall group, (**B**) prospective cohort, and (**C**) retrospective cohort.

**Figure 2:**
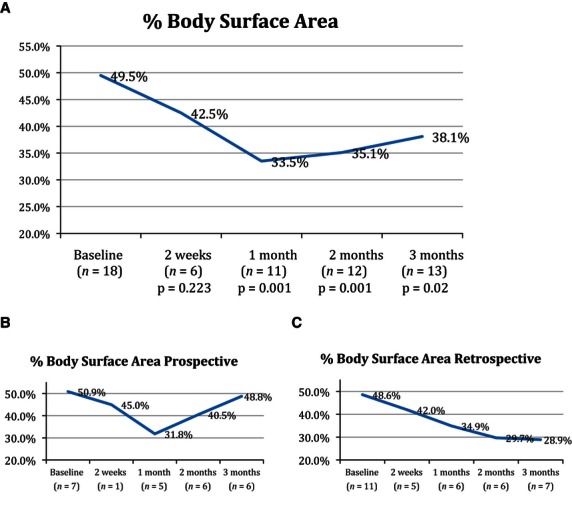
Percentage BSA affected from baseline to final visit in the (**A**) overall group, (**B**) prospective cohort, and (**C**) retrospective cohort.

Efficacy scores for the prospective and retrospective cohorts and the combined group are shown separately in Figs. 1 and 2. (The number of patients at each time point is recorded.) The mean reduction in IGA score was statistically significant at all time points using a two-tailed paired *t* test, with reductions of 1.0 at 2 weeks (p = 0.01, *n* = 6), 0.8 at 1 month (p = 0.005, *n* = 11), 0.8 at 2 months (p = 0.01, *n* = 12), and 0.9 at 3 months (p = 0.002, *n* = 13) (Fig. 1). Using the last observation carried forward (LOCF), the overall mean reduction from baseline to final measurement in all patients was 0.9 (p = 0.001, *n* = 18).

Similarly the mean reduction from baseline of BSA affected was 10.7% at 2 weeks (p = 0.22, *n* = 6), 19.0% at 1 month (p < 0.001, *n* = 11), 14.6% at 2 months (p = 0.001, *n* = 12), and 16.8% at 3 months (p = 0.01 *n* = 13). Using the LOCF, the overall mean reduction from baseline to final measurement in all patients was 14.8% (p = 0.005, *n* = 18) (Fig. 2). Although a decreasing trend was noted in the bacterial counts of many patients, it was statistically significant at 1 month only (p = 0.01, *n* = 10). No patient required oral antibiotic therapy for infective exacerbations during the course of the study.

Five patients in this feasibility study were being treated with systemic immunotherapy at the initiation of the study (four with cyclosporine and one mycophenolate mofetil). All four patients had started their cyclosporine therapy 6 to 12 weeks before enrolling in this pilot study, and the one patient taking mycophenolate mofetil had been taking it for 22 months. Clinical improvement in disease severity was observed in the four cyclosporine-treated patients, all of whom were successfully weaned off the drug during the treatment phase according to routine clinic protocol for a 6-month course. The mycophenolate mofetil-treated patient was clinically stable at the 4-week visit but was lost to follow-up thereafter.

**Figure 3:**
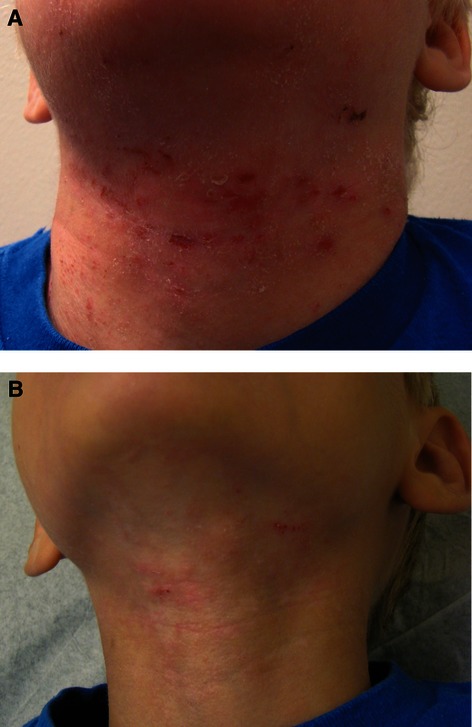
Anterior neck of the patient at (**A**) baseline and (**B**) after 10 weeks of treatment with sodium hypochlorite body wash.

**Figure 4:**
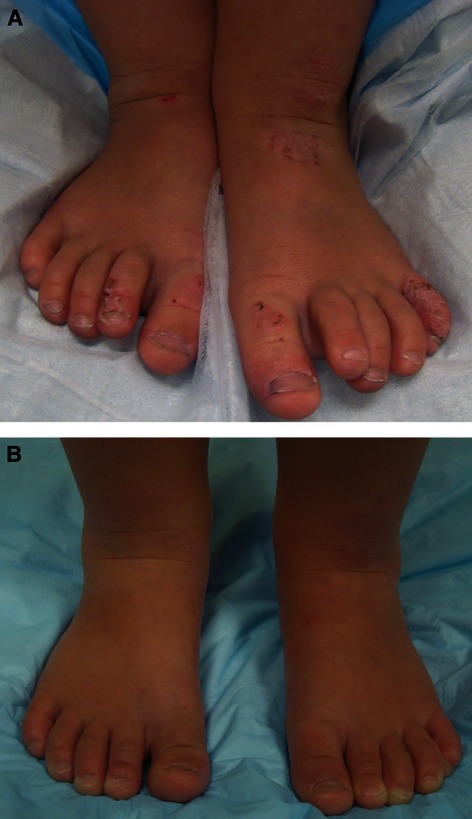
Dorsal ankles and feet of the patient (**A**) before and (**B**) after 8 weeks of treatment with sodium hypochlorite body wash.

**Figure 5:**
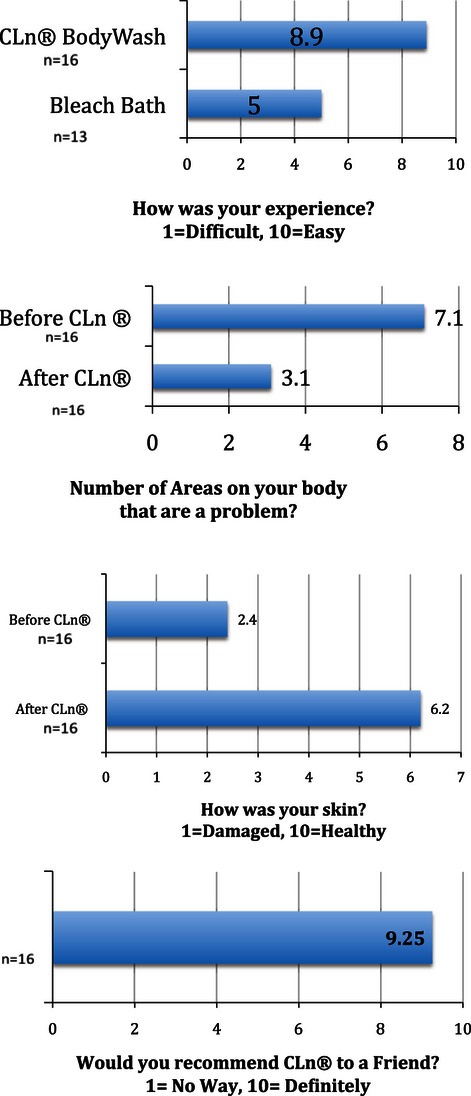
Results of patient questionnaire.

The parents of 16 of the 18 patients (89%) completed questionnaires, although four parents neglected to answer one question each. When parents were asked to rate the condition of their child's skin before and after the use of the body wash on a scale of 1 to 10 (0 = damaged and 10 = healthy), the mean score improved significantly from 2.4 (range 1–4) before treatment to 6.6 (range 3–10) after treatment (p < 0.001) (Figs. 3–5). Parents were asked to rate their experience on a scale of 1 to 10 (1 = difficult, 10 = easy). Of the 13 patients with prior use of traditional bleach baths, the mean score was 5.0 (range 2–10), compared with 8.9 (range 5–10) (p < 0.001) for the 16 patients who used the body wash. When asked whether they would recommend the body wash to friends or family on a scale of 1 to 10 (1 = no way, 10 = definitely), the mean score was 9.25 (range 7–10). The mean reported number of problematic body areas decreased from 7.1 (range 3–12) before use to 3.1 (range 0–12) after use (p < 0.001).

Three patients reported adverse events as part of the washing experience. Two of these patients reported stinging and burning, which concluded as the product was rinsed off; one did not experience an improvement in IGA or BSA and withdrew from the study after 1 month, and the other showed improvement in BSA affected (but not IGA) and completed the study. Itching occurred in one patient. This adverse event was present at 1 month, but was no longer present at 2 months; the patient showed an improvement in IGA and BSA affected and completed the study.

## Discussion

In accordance with previous studies, this study supports the high rates of *S. aureus* colonization in individuals with AD. The use of this sodium hypochlorite gel cleanser resulted in statistically significant reductions in all clinical disease severity scores after 4 weeks, with highly significant reductions from mean baseline measurements to the last observed clinical assessment.

The original traditional bleach bath study of 31 patients with moderate to severe AD compared the use of twice-weekly dilute sodium hypochlorite–containing baths (half cup of 6% bleach to a full bath of water) and nasal mupirocin ointment (for 5 consecutive days once a month) with normal baths and nasal vehicle alone for 3 months 18. Both groups were pretreated with 2 weeks of oral cephalexin. There was a significantly greater mean reduction in the Eczema Area and Severity Index (EASI) in those treated with the dilute sodium hypochlorite–containing bath and nasal mupirocin combination than in placebo-treated patients at 1 (p = 0.02) and 3 (p = 0.004) months for bath-submerged sites. Similar reductions were seen in BSA and IGA scores. The study received criticism in a subsequent review because there was a considerable difference in baseline EASI between the two treatment groups (26.9 in the treatment group vs 17.7 in the placebo group), and it was not stated whether adjustment for baseline severity was performed, raising the possibility of regression to the mean 19. Additionally, only 9 of the 15 patients in the treatment arm were followed up at 3 months. The improvements in IGA and BSA observed in the treatment arm were similar to those observed in our study.

With a greater focus on the effect of AD on disease-related quality of life, patient-friendly therapeutic interventions that minimize interference with daily living are actively being sought. Consistently high scores on the patient satisfaction questionnaire, with patients unanimously citing a marked preference for this product over traditional dilute sodium hypochlorite bleach baths, highlighted the acceptability and tolerability of this product. The use of this acceptable and convenient formulation of sodium hypochlorite may result in greater patient adherence to decolonization regimens.

Although a trend toward decreasing bacterial load was observed in our study, a crude measure was used to quantify bacterial load, the technique was not standardized, and varying sites and numbers of cultures were taken for each patient. As a result, colonization rates could not be reliably assessed. Forty-four percent of the patients with moderate to severe AD from north Texas who enrolled in this study had a positive culture for MRSA, which reflects a higher MRSA colonization rate in individuals with AD than reported in other studies 5,20,21.

Studies are currently under way to better determine the quantitative effect of body wash containing sodium hypochlorite on *S. aureus* growth using a standard or modified scrub technique, as described in the literature 22,23. This method will allow the determination of bacterial density by counting colony forming units on selective media and will use RNA sequencing for bacterial identification and sensitivities 24. In the original bleach bath study the proportion of participants with positive *S. aureus* cultures from the skin and nares was unchanged at 3 months, despite dramatic clinical improvement, suggesting that total elimination of *S. aureus* may not be necessary to decrease eczema severity. This was also suggested in a recent study that analyzed temporal shifts in the skin microbiome associated with disease flares and treatment in children with AD 25. The proportion of *S. aureus* was observed to be greater during disease flares, with successful treatment linked to greater bacterial diversity.

The antimicrobial effect of low molar ratios of sodium hypochlorite probably results from the irreversible aggregation of essential bacterial proteins 26. The safety of topical dilute sodium hypochlorite has been well established in human and animal studies 16,17. A study of the use of topical, unbuffered sodium hypochlorite for the management of burn wound infections examined the optimum concentration of sodium hypochlorite with regard to safety and efficacy 16. Minimum bactericidal concentrations were 0.006% for *S. aureus,* 0.0015% for *Streptococcus pyogenes*, and 0.003% for *Pseudomonas aeruginosa*, allowing the authors to conclude that an optimal concentration of 0.006% would be suitable for the treatment of burn wound infections without causing tissue toxicity 16. In most recommended traditional bleach baths, the concentration of sodium hypochlorite ranges from 50 parts per million (ppm; 0.005%; equivalent to 0.25 cup of bleach in one-half tub of water) to 90 ppm (0.009%; equivalent to a 0.5 cup of bleach in one-half tub of water). The concentration of sodium hypochlorite in the body wash used in the current study is 0.0061% (61 ppm), similar to the traditional dilute bleach baths, giving it optimal bactericidal activity against *S. aureus* while demonstrating excellent tolerability in patients with impaired skin barrier function.

As a pilot feasibility study, many limitations of this trial preclude us from determining conclusively the clinical response to treatment with this novel sodium hypochlorite body wash in moderate to severe AD. Our study was nonblinded, lacked a placebo arm, and was conducted in a small number of patients at a single center, and many of the retrospective patients in the initial phase of the study were lost to follow-up. Nonetheless, the dramatic clinical improvement observed in the vast majority of patients, obviating the need for oral antibiotic therapy in all patients throughout the course of the study, is promising amid a growing epidemic of multidrug-resistant *S. aureus*. Therapies formulated with dilute sodium hypochlorite provide significant advantages over several other agents for the treatment of AD, being well tolerated in the vast majority of patients and nontoxic to mucosal surfaces and lacking the potential to induce antimicrobial resistance. Larger randomized controlled studies are needed to confirm the clinical efficacy of this sodium hypochlorite–containing body wash in the treatment of moderate to severe AD. The next study will aim to quantify changes in usage of each patient's topical and systemic regimen during the course of therapy. This product may also have a valuable role for other infectious indications and in primary prevention for groups or environments at higher risk for MRSA and methicillin-sensitive *S. aureus* colonization and infections, including hospitalized patients and athletes.
